# Investigating reformulation in the Canadian food supply between 2017 and 2020 and its impact on food prices

**DOI:** 10.1017/S136898002400226X

**Published:** 2024-11-22

**Authors:** Emily R Ziraldo, Guanlan Hu, Ayesha Khan, Mary R L’Abbé

**Affiliations:** Department of Nutritional Sciences, Temerty Faculty of Medicine, University of Toronto, Toronto, ON, Canada

**Keywords:** Food price, Nutrient profiling, Food composition database, Nutritional composition, Food supply

## Abstract

**Objective:**

This study examined the relationship between reformulation and food price in Canadian packaged foods and beverages between 2017 and 2020.

**Design:**

Matched foods and beverages in the University of Toronto Food Label Information and Price 2017 and 2020 databases were analysed (*n* 5774). Price change by food category and by retailer were compared using Wilcoxon signed-rank tests. The proportion of products with changes in calories and nutrient levels were determined, and mixed-effects models were used to examine the relationship between reformulation and price changes. The Food Standards Australia New Zealand (FSANZ) nutrient profiling model was applied to calculate nutritional quality scores, and mixed-effects models were used to assess if changes in nutritional quality score were associated with price changes.

**Setting:**

Large grocery retailers by market share in Canada.

**Participants:**

Foods and beverages available in 2017 and 2020.

**Results:**

Food price changes differed by retailer and by food category (e.g. increased in Bakery, Snacks, etc; decreased in Beverages, Miscellaneous, etc.). Nutrient reformulation was minimal and bidirectional with the highest proportion of products changing in sodium (17·8 %; 8·4 % increased and 9·4 % decreased). The relationship between nutrient reformulation and price change was insignificant for all nutrients overall and was not consistent across food categories. Average FSANZ score did not change (7·5 in both years). For Legumes and Combination dishes, improvements in nutritional quality were associated with a price decrease and increase, respectively.

**Conclusions:**

Stronger policies are required to incentivise reformulation in Canada. Results do not provide evidence of reformulation impacting food prices.

A global shift in dietary patterns towards increased consumption of energy-dense foods and diets high in sodium, sugar and saturated fat and low in fruit, vegetables and whole grains, accompanied by sedentary lifestyles, overconsumption of alcohol and tobacco use has contributed to the rising prevalence of non-communicable diseases worldwide^(1,2)^.

To improve diet quality and reduce the prevalence of non-communicable diseases in Canada, the federal government launched the Healthy Eating Strategy in 2016 with the aim of making it easier for Canadians to choose healthier foods through a suite of food policies that improve healthy eating information and the nutritional quality of food^(3)^. Policies under the Healthy Eating Strategy include voluntary sodium reduction targets for processed foods, changes to the Nutrition Facts table, an updated Canada’s Food Guide (2019) and mandatory front-of-package labelling regulations, among others^(3)^.

Food policies have the potential to stimulate reformulation. Food and beverage reformulation refers to changing the nutrient composition of a product with the aim of improving the nutritional quality without compromising desirable product attributes (e.g. flavour and texture)^(4)^. A review on the impact of reformulation found that consumers usually accept and purchase reformulated products and that reformulation can improve nutritional intakes^(5)^. Additionally, there is evidence for positive health impacts with studies on *trans* fat reformulation finding reduced risk for CVD^(5)^ and one study on sodium reduction finding a positive effect on blood pressure^(6)^. The majority of reformulation policies and empirical scientific evidence on reformulation have focused on sodium and *trans* fat; however, sugar and saturated fat are also nutrients of concern that may be targets for reformulation policies^(7)^. Due to its cost-effectiveness, the WHO has named sodium reformulation as a ‘best buy’ population-level intervention to improve diets and prevent and control non-communicable diseases^(8)^. Reformulation is considered an equitable approach, as consumer behaviour change is not required to benefit^(4,9)^. Reformulation after regulatory change in Canada was previously seen when industry voluntarily reduced *trans* fats in products following the implementation of a mandatory *trans* fat declaration on the Nutrition Facts table in 2003^(10)^. Subsequently, regulations prohibiting partially hydrogenated oils, the largest source of industrially produced *trans* fats, came into force in 2018^(3)^, further reducing *trans* fats in Canadian foods. However, unlike the regulations for *trans* fats, Healthy Eating Strategy policies generally provide weaker incentives for reformulation, for example, adherence to the sodium reduction targets is voluntary, current food labelling policies leave the decision to reformulate and the magnitude of reformulation at the discretion of food manufacturers, Canada’s Food Guide (2019) targets consumer behaviour change by providing dietary guidance rather than manufacturer reformulation and the mandatory front-of-package labelling regulations which may incentivise reformulation do not come into force until January 2026^(3)^.

However, companies have long raised that policies that encourage reformulation may result in increased prices of healthier, reformulated foods relative to less healthy options^(11)^ due to the investment required for manufacturers to reformulate^(12)^ or substitutions for ingredients that are more expensive^(13–15)^. Following the *trans* fat labelling changes in 2003, Ricciuto *et al.* (2009) found that margarines with lower *trans* fat content were more expensive than margarines with higher amounts of *trans* fats^(16)^. This relationship was stronger post-labelling changes, suggesting that reformulation was restricted to higher-priced margarines^(16)^. This is of concern as price is an important factor in food purchasing decisions^(17)^, particularly for lower income consumers^(18)^. If foods reformulated to be healthier are more expensive, it will create a barrier to consumers who are looking to make healthier choices, and notably, the potential population health benefits of the Healthy Eating Strategy will be diminished overall, and inequitably achieved by socio-economic status gradient, exacerbating existing nutrition inequities in Canada^(19)^.

The most recent assessments of reformulation in Canada were focused on sodium and sugar and used data collected in 2013 and 2017^(20,21)^. Updated, comprehensive analysis, that includes evaluation of price changes associated with reformulation, is needed to assess progress towards improving the nutritional quality of foods under the Healthy Eating Strategy. Therefore, the objective of this study was to examine the relationship between reformulation and food price in a large sample Canadian packaged foods and beverages between 2017 and 2020.

## Materials and methods

### Database

This study analysed data from the University of Toronto Food Label Information and Price (FLIP) 2017 (FLIP2017; *n* 19 720) and 2020 (FLIP2020; *n* 74 445) databases, details for which have been previously published^(22,23)^. Briefly, FLIP contains label information (e.g. product name, brand, Nutrition Facts table, ingredients, price, universal product code (UPC), retailer-specific ID, etc.) for branded foods and beverages sold at large grocery retailers by market share in Canada. FLIP2017 was collected in store using a mobile app between May and September 2017 from the three largest retailers in Canada (approximately 68 % of grocery retail sales)^(22)^. FLIP2020 was collected by web-scraping seven grocery retailer websites (approximately 80 % of grocery retail market share) between May 2020 and February 2021, including the same retailers from FLIP2017, and using optical character recognition to read label images^(23)^. Both FLIP2017 and FLIP2020 included all container sizes for a food or beverage. FLIP2020 includes all foods available on the retailer website; however, FLIP2017 did not include fresh or unpackaged foods due to the lack of on-package nutrition information. Products in FLIP were classified into Health Canada’s Table of Reference Amounts (TRA) food categories, which includes major (e.g. Bakery and Dairy) and minor categories (e.g. bread, muffins, bagels; cottage cheese, hard cheese, milk, cream)^(24)^.

### Data preparation

**Fig. 1:**
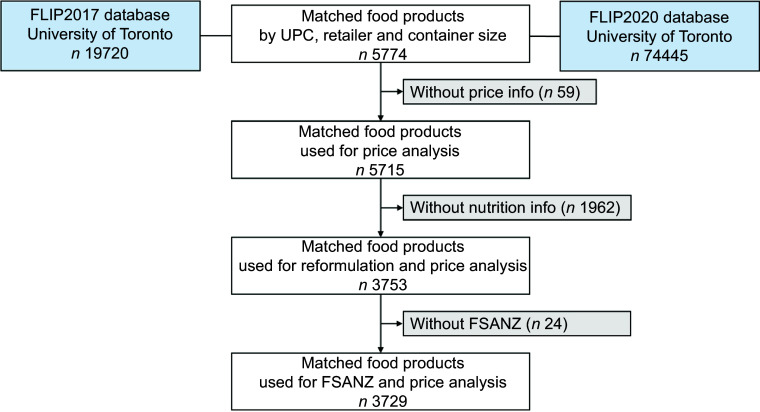
Data preparation flow chart for matching food products, analysing food prices and examining the relationship between reformulation, nutrition quality and price. The validated FSANZ nutrient profiling system, which considers nutrients to limit, nutrients to encourage and food components and is used in Australia and New Zealand to determine a product’s eligibility to carry a health claim, was used to calculate a nutritional quality score for food products^(25)^. FLIP, Food Label Information and Price; FSANZ, Food Standards Australia New Zealand; UPC, universal product code.

Products in the FLIP2017 and FLIP2020 databases were matched by UPC and retailer-specific ID, retailer and container size (*n* 5774), as shown in Fig. 1. Products in TRA food categories W. Food for children <4 years old (*n* 100 matched products) and X. Meal replacements and substitutes (*n* 12) were excluded as they are subject to specific regulations regarding nutrient content in Canada. The price per 100 g (or ml) and nutrients per 100 g (or ml) were calculated using container and serving sizes, respectively. Product brands were categorised into four types, including private label premium, private label discount, multinational, and domestic or other. For price analysis, products without valid price information (i.e. either missing data or unclear the quantity for which the price related) were excluded, leaving 5715 matched products for analysis. Matched products with complete nutrition information (*n* 3753) were used for reformulation analysis.

We further determined whether and to what extent reformulation of nutrients (calories, carbohydrates, protein, fat, saturated fat, sodium and sugar) occurred by calculating the nutrient change per 100 g (or ml) between 2020 and 2017 in the matched products. Matched food products were categorised into five reformulation groups based on the degree of nutrient changes per 100 g (or ml) using Health Canada’s labelling thresholds of 15 % of the Daily Value (a lot) and 5 % (a little)^(26)^. The five reformulation groups were as follows: (1) large decrease (≥–15 %), (2) medium decrease (–5 % to –14·9 %), (3) little change (–4·9 % to +4·9 %), (4) medium increase (+5 % to +14·9 %) and (5) large increase (≥+15 %). Price changes between 2020 and 2017 per 100 g (or ml) for matched products were calculated.

We applied the Food Standards Australia New Zealand (FSANZ) nutrient profiling system, which takes into account both nutrients to limit, nutrients to encourage and food components (i.e. fruit, vegetables, nuts and legumes), to calculate a nutritional quality score for packaged foods and beverages^(25)^. Foods were then categorised into the three FSANZ food categories and assessed against the Nutrient Profiling Scoring Criteria (NPSC), including Category 1 – Beverages (FSANZ score <1 meets NPSC), Category 3 – Cheese or processed cheese (with calcium content >320 mg/100 g), edible oil, edible oil spread, margarine, butter (FSANZ score <28 meets NPSC), and Category 2 – Other foods that are not included in Category 1 or 3 (FSANZ score <4 meets NPSC). The NPSC criteria are used to determine if a product is eligible to carry a health claim in Australia and New Zealand. A lower FSANZ score indicates higher product healthfulness. Foods that were not eligible for FSANZ calculation or were missing needed data were further excluded and 3729 matched products remained for the FSANZ and price relationship analysis.

### Statistical analyses

Descriptive statistics including the mean and median for central tendency and standard deviation for dispersion were calculated for the distribution of price and FSANZ scores by retailer, TRA food categories and FSANZ food categories. Wilcoxon signed-rank tests were used to compare the price and nutritional quality (overall and for each category) between the 2017 and 2020 matched food products. The proportion of products in each reformulation group (large decrease, medium decrease, little change, medium increase and large increase) by nutrient (calories, carbohydrates, protein, fat, saturated fat, sodium and sugar) were calculated. Chi-square tests were conducted to assess the association between reformulation groups and brand types. Mixed-effects models, adjusted for retailer (random effect), container size and brand type, were fit to assess whether reformulation of each nutrient was associated with price change in the matched products. The little change (±4·9 %) reformulation group was set as the reference, and groups with a sample size <5 were excluded from model fitting. Mixed-effects models, adjusted for retailer, container size and brand type, were used to analyse the association between FSANZ score change and price change. To account for multiple testing within the same sample, *P*-values were adjusted using the Benjamini–Hochberg procedure. All analyses were conducted using R version 4.2.1.

This analysis did not adjust for inflation for various reasons. For the comparison of prices between 2017 and 2020, we were interested in price changes as seen by consumers, which would include price changes due to inflation. This also allowed for comparison to previously reported food price changes over this time period^(27)^. For analysis of food price changes associated with reformulation, we compared price changes between reformulation groups. As inflation is expected to equally impact all reformulation groups, price changes due to inflation would cancel out when the price change of each group was compared against the reference group (i.e. little change). For the analysis of the association between change in FSANZ score and change in price, inflation was anticipated to be similar within each TRA food category (e.g. Bakery and Beverages) as similar products were grouped together and if products are impacted equally by inflation, the association observed would reflect price changes due to other factors (e.g. changes in nutritional quality). In addition, previous data have found that between 2017 and 2020, food inflation rates in Canada were low, between 0·1 and 2·8 %^(27)^.

## Results

### Food price change of matched products between 2017 and 2020

**Fig. 2:**
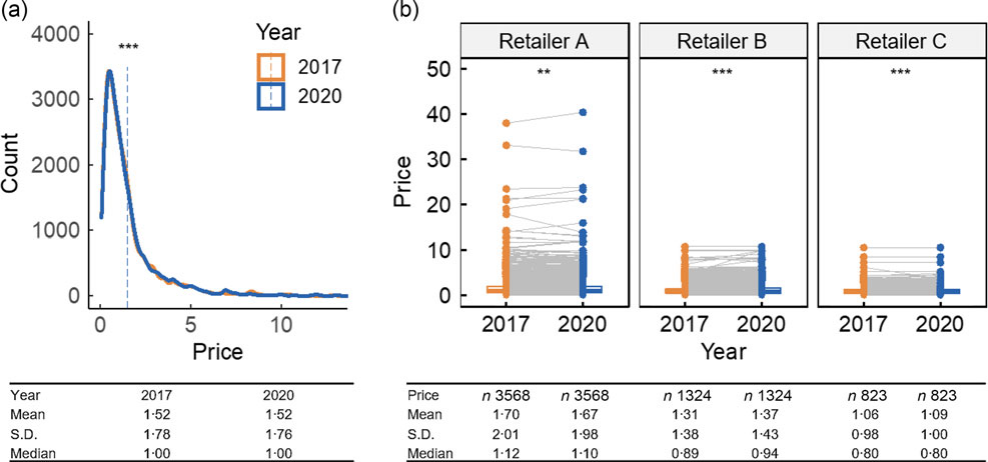
Food price change ($/100 g (or ml)) between FLIP2017 and FLIP2020 matched products (*n* 5715) (a) overall and (b) by grocery retailer. Products were matched by product code (UPC or retailer-specific ID), retailer and container size. Wilcoxon signed-rank tests were used to compare undiscounted prices of matched products across years. Significance levels: ***P < 0·001, **P < 0·01. FLIP, Food Label Information and Price; UPC, universal product code.

The price of products matched between 2017 and 2020 from Canadian major food retailers did not change overall (mean and s d were $1·52 ± 1·78/100 g (or ml) in 2017 and $1·52 ± 1·76 in 2020, the median in both years was $1·00/100 g (or ml)) (Fig. 2). However, there were significant changes by TRA food category (e.g. increased in A. Bakery, G. Eggs, I. Fish, J. Fruit, K. Legumes, L. Meat, Q. Salad, S. Snacks, T. Soups and V. Vegetables (*P* < 0·05); decreased in B. Beverages, M. Miscellaneous, U. Sugars and W. Foods for children (*P* < 0·05), etc; see online supplementary material, Supplementary Table 1). Price trends between 2017 and 2020 also differed by retailer (Fig. 2(b) and see online supplementary material, Supplementary Table 1); the price of nearly all TRA food categories increased in two food retailers (Retailers B and C), while the other major retailer (Retailer A) showed different price trends for some TRA food categories. For example, the mean price in TRA food category N. Combination Dishes significantly decreased for Retailer A but increased for Retailers B and C.

### Nutrition reformulation and food price change

Table 1 reports the results of the mixed-effects models for change in price between 2017 and 2020 by reformulation group. Between 2017 and 2020, most products (82·2–90·6 %) had little change in calories or nutrient levels. The highest proportion of products changed in sodium level (17·8 %); however, similar proportions of products decreased (5·0 % large decrease; 4·4 % medium decrease) and increased (5·1 % large increase; 3·3 % medium increase) in sodium content. This was also observed for calories and all other nutrients, with similar proportions of products in the large decrease and large increase groups and in the medium decrease and medium increase groups. After *P*-values were adjusted for multiple testing, there were no significant differences in price changes between products that had little change in nutrient levels compared with products that had increased or decreased (*P* ≥ 0·05 for all comparisons).

**Table 1.** Relationship between food price and nutrient reformulation in matched products, (*n* 3753 matched products) by nutrient-level changes^*^

Reformulation group^†^	Calories				Carbohydrates				Protein				Fat			
	*n*	%	*β*	95 % CI	*n*	%	*β*	95 % CI	*n*	%	*β*	95 % CI	*n*	%	*β*	95 % CI
Large decrease	95	2·5	0·054	–0·013, 0·122	97	2·6	0·007	–0·059, 0·073	133	3·5	0·010	–0·046, 0·067	108	2·9	0·052	–0·010, 0·115
Medium decrease	140	3·7	0·026	–0·029, 0·082	119	3·2	–0·001	–0·061, 0·059	33	0·9	0·111	0·001, 0·222	92	2·5	0·064	–0·003, 0·133
Little change	3305	88·1	ref	ref	3341	89·0	ref	ref	3399	90·6	ref	ref	3327	88·6	ref	ref
Medium increase	123	3·3	0·026	–0·033, 0·086	96	2·6	0·064	–0·002, 0·130	42	1·1	0·053	–0·046, 0·155	64	1·7	0·057	–0·025, 0·139
Large increase	90	2·4	0·016	–0·052, 0·085	100	2·7	0·005	–0·060, 0·071	146	3·9	–0·013	–0·068, 0·042	162	4·3	0·016	–0·036, 0·068

	Saturated fat				Sodium				Sugar							
Reformulation group^†^	*n*	%	*β*	95 % CI	*n*	%	*β*	95 % CI	*n*	%	*β*	95 % CI				
Large decrease	168	4·5	0·021	–0·029, 0·072	189	5·0	0·000	–0·048, 0·050	161	4·3	0·043	–0·009, 0·095				
Medium decrease	24	0·6	0·101	–0·030, 0·233	165	4·4	0·050	–0·001, 0·103	50	1·3	–0·017	–0·108, 0·075				
Little change	3340	89·0	ref	ref	3084	82·2	ref	ref	3315	88·3	ref	ref				
Medium increase	24	0·6	0·065	–0·066, 0·197	122	3·3	–0·006	–0·064, 0·054	64	1·7	–0·002	–0·082, 0·078				
Large increase	197	5·2	0·018	–0·029, 0·067	193	5·1	–0·008	–0·055, 0·040	163	4·3	0·023	–0·028, 0·074				

%, proportion of matches by reformulation group; n, sample size; ref, reference group.

* *β* values for the effect size and CI were obtained by fitting mixed-effects models for price change and nutrient reformulation category per 100 g (or ml), adjusted for retailer, brand type and container size, and reference group is the little change group. All *P*-values were adjusted for multiple comparisons using the Benjamini–Hochberg procedure and were not significant (*P* > 0.05).

† Products were categorised into five reformulation groups based on the degree of calorie or nutrient changes per 100 g (or ml) between 2017 and 2020 using Health Canada’s labelling thresholds of 15 % of the Daily Value (a lot) and 5 % (a little) as cut-offs^(26)^. The five reformulation groups were as follows: (1) large decrease (≥–15 %), (2) medium decrease (–5 % to –14.9 %), (3) little change (–4.9 % to +4.9 %), (4) medium increase (+5 % to +14.9 %) and (5) large increase (≥+15 %).

**Fig. 3:**
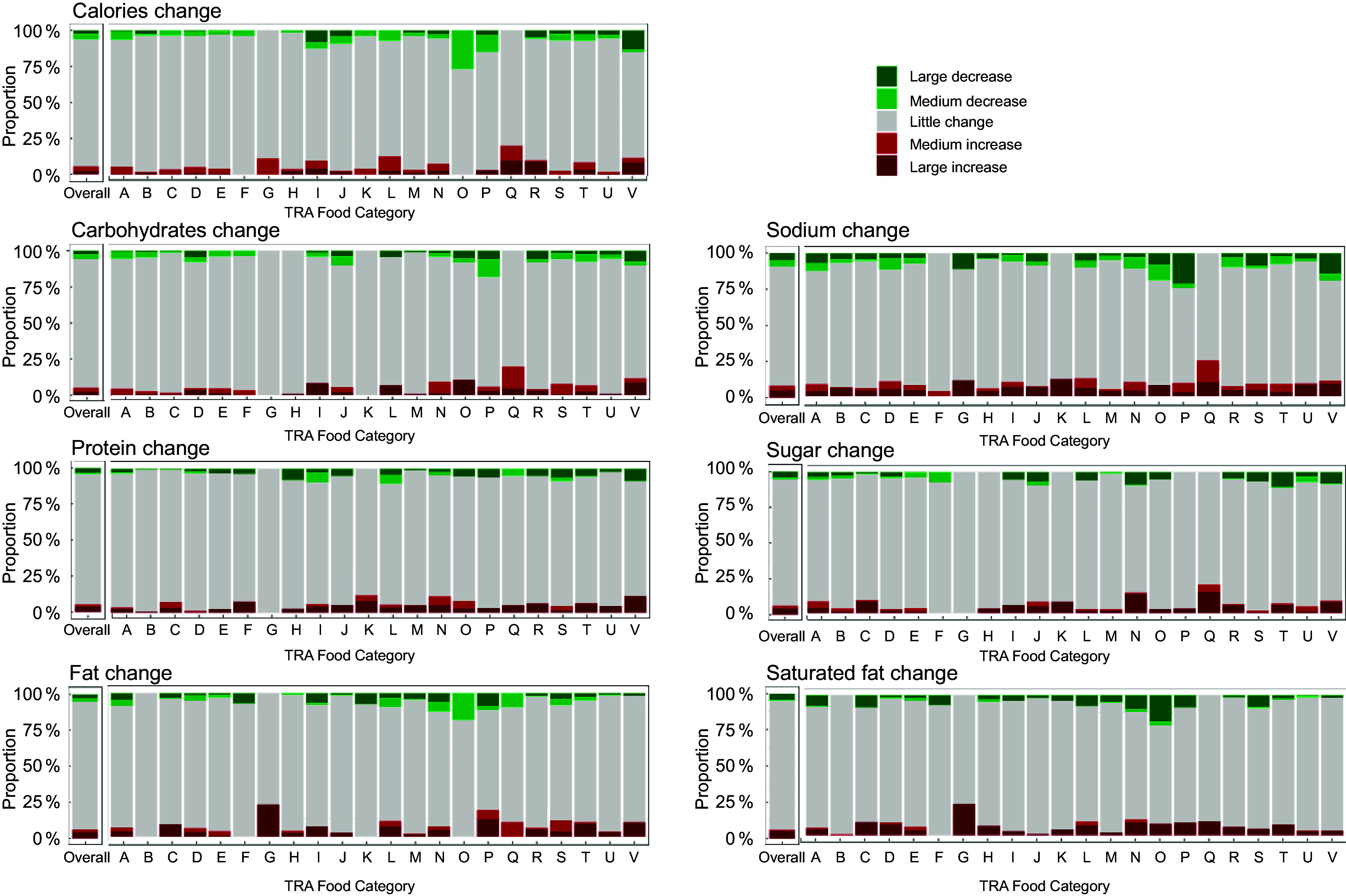
The proportion of matched products that changed in calories and nutrient levels between 2017 and 2020 overall and by TRA food category (*n* 3753). Products were matched by product code (UPC or retailer-specific ID), retailer and container size, and only products with complete nutrition information were included. Products were categorised into five reformulation groups based on the magnitude and direction of calorie or nutrient changes per 100 g (or ml) between 2017 and 2020 using Health Canada’s labelling thresholds of 15 % of the Daily Value (a lot) and 5 % (a little) as cut-offs^(26)^. The five reformulation groups were as follows: (1) large decrease (≥–15 %), (2) medium decrease (–5 % to –14·9 %), (3) little change (–4·9 % to +4·9 %), (4) medium increase (+5 % to +14·9 %) and (5) large increase (≥+15 %). TRA food categories^(24)^: A. Bakery, B. Beverages, C. Cereals and other grains, D. Dairy products and substitutes, E. Desserts, F. Dessert toppings and fillings, G. Eggs and substitutes, H. Fats and oils, I. Marine and fresh water fish, J. Fruit and fruit juices, K. Legumes, L. Meat and substitutes, M. Miscellaneous, N. Combination dishes, O. Nuts and seeds, P. Potatoes, sweet potatoes and yams, Q. Salads, R. Sauces, dips, gravies and condiments, S. Snacks, T. Soups, U. Sugars and sweets and V. Vegetables. TRA, Table of References Amounts; UPC, universal product code.

See online supplementary material, Supplementary Table 2 and Fig. 3 show the number and proportion of products reformulated between 2017 and 2020 by TRA food category. For calories, O. Nuts and seeds had the highest proportion of reformulated products (27·0 %, *n* 10). By reformulation group, V. Vegetables had the highest proportion of products with a large decrease in calories (13·1 %, *n* 30), O. Nuts and seeds for medium decrease (27·0 %, *n* 10) and Q. Salads for both medium increase (10·0 %, *n* 2) and large increase (10·0 %, *n* 2).

For saturated fat, sodium and sugar (see online supplementary material, Supplementary Table 2 and Fig. 3), the categories with the highest proportion of reformulated products were O. Nuts and seeds (29·7 %, *n* 11), P. Potatoes, sweet potatoes and yams (33·3 %, *n* 11) and N. Combination dishes (24·3 %, *n* 52). The categories with the highest proportion of products that decreased (either a large or medium decrease) in saturated fat, sodium and sugar content were O. Nuts and seeds (21·6 %, *n* 8), P. Potatoes, sweet potatoes and yams (24·2 %, *n* 8), and T. Soups (11·3 %, *n* 24). The categories with the highest proportion of products that increased (either a large or medium decrease) in saturated fat, sodium and sugar content were N. Combination dishes (11·2 %, *n* 24), Q. Salads (25·0 %, *n* 5) and Q. Salads (20·0 %, *n* 4).

The results from the mixed-effects model for the association between change in price between 2017 and 2020 and reformulation group by TRA food category are shown in see online supplementary material, Supplementary Table 2. For sodium, in TRA food category C. Cereals and other grains, products that had a large increase in sodium were associated with a greater decrease in price relative to the little change group (*β* = –0·13, 95 % CI (–0·22, –0·03)) and products that had a large decrease in sodium were associated with a greater increase in price (*β* = 0·14, 95 % CI (0·04, 0·23)). Similarly, in TRA food category L. Meat and substitutes, both a medium (*β* = –0·33, 95 % CI (–0·54, –0·13)) and large (*β* = –0·25, 95 % CI (–0·46, –0·04)) increase in sodium were associated with a larger decrease in price relative to the little change group, while products that had a medium decrease in sodium increased in price more (*β* = 0·36, 95 % CI (0·12, 0·60)) than the little change group. However, when stratified by TRA food category, sample sizes were small in most reformulation groups and no clear, consistent association was observed between price change and reformulation group across TRA food categories.

For calories and all nutrients, results of *χ*^2^ tests to assess interaction between brand type and reformulation group, devoid of price, showed a statistically significant association. This indicates that reformulation group depends on product brand type (i.e. private label premium, private label discount, multinational, and domestic or other). As this study focused on the association between reformulation and prices changes, no further statistical testing comparing brand types was performed. However, the contingency tables showed private label premium as the brand type with the highest proportion of products in any reformulation group for energy and all nutrients (15·4–27·5 % in a reformulation group), while private label discount had lower proportions of products in reformulation groups (5·9–10·5 %). Among multinational and domestic and other brands, 6·9–15·8 % and 8·4–15·5 % of products were in a reformulation group.

### The relationship between healthfulness and food price change

**Fig. 4:**
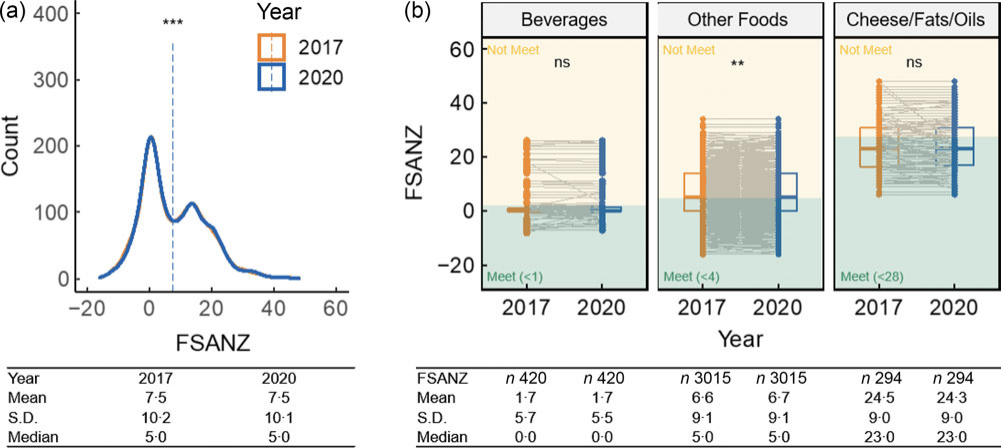
FSANZ score change between FLIP2017 and FLIP2020 matched products (*n* 3729), (a) overall and (b) by FSANZ food category. Products were matched by product code (UPC or retailer-specific ID), retailer and container size, and only products with valid nutrition information and FSANZ scores were included. Wilcoxon signed-rank tests were used to compare FSANZ scores across years. FSANZ, a validated nutrient profiling system used in Australia and New Zealand to determine a product’s eligibility to carry a health claim, was applied to calculate a nutritional quality score for food products^(25)^. Significance levels: ***P < 0·001, **P < 0·01. FLIP, Food Label Information and Price; FSANZ, Food Standards Australia New Zealand; UPC, universal product code.

Figure 4(a) compares the 2017 and 2020 distributions of FSANZ scores for all matched products. The mean and median FSANZ scores in 2017 and 2020 were the same (mean and s d were 7·5 (10·2) in 2017 and 7·5 (10·1) in 2020, the median in both years was 5·0). Within the FSANZ food categories, there was a statistically significant but minor increase in FSANZ scores (indicating that products became less healthy) over time in the Other foods category (2017 mean (s d) = 6·6 (9·1), 2020 mean (s d) = 6·7 (9·1), *P* < 0·01; Fig. 4(b)). There was no significant change in FSANZ score for the Beverages or Cheese/Fats/Oils categories.

**Fig. 5:**
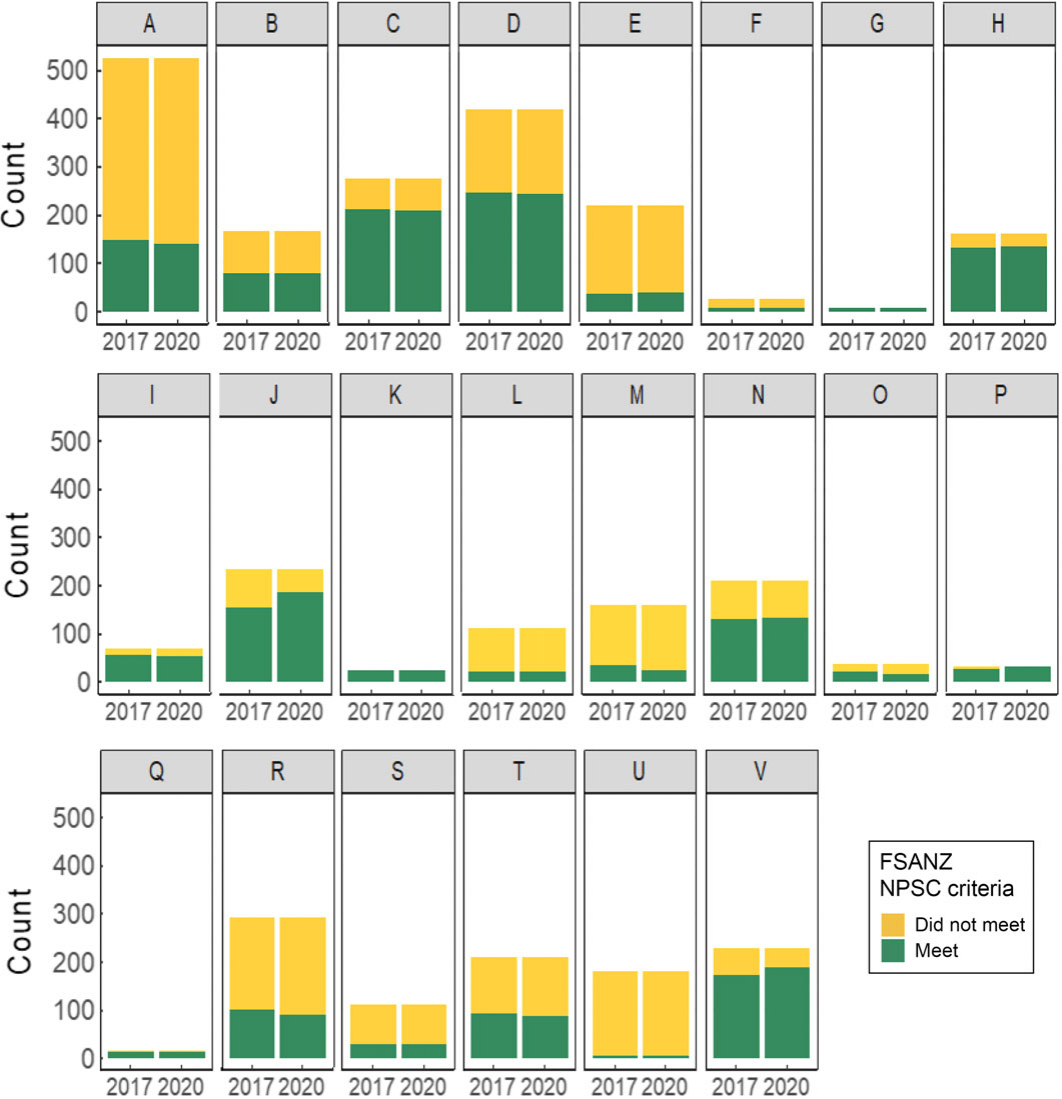
The number of foods and beverages in FLIP2017 and FLIP2020 (*n* 3729) that met the FSANZ NPSC health claims criteria^(25)^ by TRA food category. The same foods and beverages, matched by product code (UPC or retailer-specific ID), retailer and container size, were evaluated for FLIP2017 and FLIP2020. TRA food categories^(24)^: A. Bakery, B. Beverages, C. Cereals and other grains, D. Dairy products and substitutes, E. Desserts, F. Dessert toppings and fillings, G. Eggs and substitutes, H. Fats and oils, I. Marine and fresh water fish, J. Fruit and fruit juices, K. Legumes, L. Meat and substitutes, M. Miscellaneous, N. Combination dishes, O. Nuts and seeds, P. Potatoes, sweet potatoes and yams, Q. Salads, R. Sauces, dips, gravies and condiments, S. Snacks, T. Soups, U. Sugars and sweets and V. Vegetables. FLIP, Food Label Information and Price; FSANZ, Food Standards Australia New Zealand; NPSC, Nutrient Profiling Scoring Criterion; TRA, Table of Reference Amounts; UPC, universal product code.

Figure 5 compares the count of 2017 and 2020 products in each TRA food category meeting the FSANZ NPSC health claims criteria. The proportion of products meeting the health claims criteria in 2017 and 2020 was the same over time in most food and beverage categories. Categories J. Fruit and fruit juices and P. Potatoes, sweet potatoes and yams experienced the largest increases (+12·7 % and +12·1 %) in products meeting the health claims criteria (from 66·1 % in 2017 to 78·8 % in 2020 for category J and from 84·8 % to 97·0 % for category P). Conversely, a large decrease in the proportion of products meeting the criteria was observed in category O. Nuts and seeds (62·1 % in 2017 to 43·2 % in 2020).

Table 2 reports the results of the mixed-effects models fit for the relationship between food price and FSANZ score change in matched 2017 and 2020 products by TRA food category. In the overall sample, a change in FSANZ score did not significantly predict food price change. However, a decrease in the healthfulness of a product over time (a 1-unit FSANZ score increase) was significantly associated with a $0·054 increase in food price per 100 g (or ml) (95 % CI (0·024, 0·083)) in the K. Legumes category and with a price decrease of $0·021 per 100 g (or ml) (95 % CI (–0·037, –0·005)) in N. Combination dishes.

**Table 2.** Relationship between food price and FSANZ score change in matched products by TRA food category^†^

TRA food category	*n*	*β*	95 % CI
Overall	3729	0·001	–0·004, 0·006
A. Bakery	525	0·014	0·000, 0·028
B. Beverages	168	–0·017	–0·074, 0·040
C. Cereals and other grains	277	–0·003	–0·026, 0·020
D. Dairy products and substitutes	420	–0·005	–0·015, 0·006
E. Desserts	220	0·015	–0·014, 0·045
F. Dessert toppings and fillings	27	0·000	–0·044, 0·045
H. Fats and oils	163	–0·004	–0·024, 0·016
I. Marine and fresh water animals	71	0·006	–0·100, 0·119
J. Fruit and fruit juices	236	–0·004	–0·010, 0·002
K. Legumes	25	0·054**	0·024, 0·083
L. Meat and substitutes	112	–0·001	–0·032, 0·029
M. Miscellaneous	160	–0·046	–0·107, 0·026
N. Combination dishes	212	–0·021*	–0·037, –0·005
O. Nuts and seeds	37	0·004	–0·004, 0·012
P. Potatoes, sweet potatoes and yams	33	0·001	–0·002, 0·005
Q. Salads	14	0·025	–0·021, 0·072
R. Sauces, dips, gravies and condiments	291	0·000	–0·021, 0·022
S. Snacks	111	0·015	–0·004, 0·033
T. Soups	210	–0·007	–0·026, 0·011
U. Sugars and sweets	179	0·002	–0·024, 0·029
V. Vegetables	229	–0·001	–0·012, 0·010

FSANZ, Food Standards Australia New Zealand; TRA, Table of Reference Amounts; n, sample size; UPC, universal product code.

† Food products matched by same ID (UPC and retailer-specific product number), retailer and container size. Mixed-effects models for price change and FSANZ score change per 100 g (or ml), adjusted for retailer, container size and brand type. Significance level: **P < 0.01, *P < 0.05. TRA food category G. Eggs and substitutes not included due to low sample size (*n* 9).

## Discussion

This study provides the first comprehensive analysis of the relationship between reformulation and food price in Canadian packaged foods and beverages over time. Overall, food prices changed very little between 2017 and 2020. In addition, few foods and beverages changed in nutrient levels and, of reformulated products, similar amounts were reformulated to be healthier and less healthy. When reformulation did occur, it was not consistently associated with price changes. Our findings indicate that Canadian food policies in place between 2017 and 2020, which encouraged voluntary reformulation, were not effective at improving the nutritional quality of foods and beverages. Where reformulation occurred, we did not find evidence of reformulation impacting prices in most food categories.

In general, food prices are expected to increase over time due to inflation. Our finding that there was little change in prices aligns with Canadian food inflation data showing low inflation rates (range: –0·1 %–2·8 %) between the FLIP2017 and FLIP2020 collections (May 2017–February 2021)^(27)^. We also found that for two retailers and most food categories, mean prices were higher in 2020 than 2017. There were some exceptions, particularly for Retailer A in which some food categories had a lower mean price in 2020 (e.g. Beverages, Desserts, Fats and Oils). In general, the price of retail food items reflects various steps in the supply chain, including production, packaging, processing, distribution, other marketing costs and competitive factors. Differences in price trends between retailers emphasise the role of retailers in setting food prices and suggest that retailers use varied pricing strategies. Although the food retail sector appears highly competitive, there are opportunities for food retailers to set prices that exceed their marginal costs^(28)^. Retailers role in food pricing has been a topic of discussion recently in Canada^(29–31)^ and led to the recent development of a Grocery Code of Conduct intended to ‘enhance transparency, predictability and fair dealing’^(32)^ in the grocery supply chain.

Results also suggest that there has been very little reformulation of packaged foods and beverages in the Canadian food supply between 2017 and 2020 and, when reformulation has occurred, it has been bidirectional, with similar proportions of products increasing and decreasing in nutrient content or healthfulness score. Previous assessments of sugar and sodium reformulation in Canada have reported similar results. Between 2013 and 2017, 76·6 % of foods and beverages did not change in sugar content and of products that did change, similar proportions increased (11·0 %) and decreased (12·4 %)^(20)^. In this study, we found that between 2017 and 2020, a higher proportion of products (88·3 %) did not change or changed only a little (<5 %) in sugar content, while 6·0 % increased and 5·6 % decreased ≥ 5 %. The difference in reformulation proportions between time periods is likely attributable to the use of a threshold (±5 %) in this analysis. For sodium, assessments of progress towards achieving Health Canada’s sodium reduction targets have shown that industry voluntary efforts to reduce sodium in the food supply have been underwhelming and reformulation has been minimal^(21,33)^. Our finding that reformulation was bidirectional suggests that reformulation occurs for reasons other than improving nutritional quality, for example, ‘skimpflation’ is when a manufacturer reformulates its product with cheaper ingredients^(34)^. Overall, the results from this paper support the need for stronger policies to incentivise the food industry to reduce sodium levels – as has been previously noted^(35)^. Voluntary sodium reduction targets, when accompanied by other initiatives, can be effective at reducing sodium intakes. This was observed through the successful voluntary salt reduction strategy implemented in the United Kingdom in which voluntary sodium reduction targets were accompanied by a consumer awareness campaign, efforts to engage industry to reformulate, threats of regulation and other policy initiatives^(36)^. Mandatory policies also have greater impacts on reformulation with reductions in sodium intakes reported following the implementation of mandatory sodium reduction targets in Argentina^(37)^ and South Africa^(38)^ as well as reformulation observed in Chile following enactment of the Law of Food Labelling and Advertising which included mandatory front-of-package warning labels and restrictions on marketing unhealthy foods and beverages^(39)^.

We did not find an association between nutrient reformulation and price changes overall. For two food categories, C. Cereals and other grains and L. Meat and substitutes, a notable a trend was that relative to products with little change in sodium, products reformulated to be lower sodium had a larger *increase* in price and products reformulated to be higher sodium had a larger *decrease* in price. This association was not consistent or in a uniform direction across other food categories, with N. Combination dishes showing the opposite relationship – products that had a medium increase in sodium had a larger *increase* in price relative to the little change group. Similar inconsistencies in food prices between food categories have been observed in other Canadian studies^(40,41)^, indicating the complexity of food prices and the importance of category-level analysis when conducting food price research. As minimal reformulation was identified in this study, the category-level results for reformulation and food price change should be interpreted with caution, due to the small sample sizes in most reformulation groups.

When reformulation was assessed using FSANZ scores, an increase in healthfulness was significantly associated with an increase in price in only food category N. Combination Dishes. The opposite relationship was observed in food category K. Legumes in which an increase in healthfulness was significantly associated with a decrease in price. These results contrast food industry feedback to policies and regulations that encourage reformulation, which has been that reformulation is costly and will be reflected in higher prices for consumers^(12)^. We observed that for most food categories, reformulation did not impact food prices. Similar conclusions were seen in a recent evaluation of the impact of the 2016 Chilean Law of Food Labelling and Advertising, in which there was no association between reformulation and food prices, despite extensive reformulation occurring^(42)^. Overall, the implications of these findings are important for future food policy development as they call into question the commonly accepted cost barrier to reformulation.

The use of two large, branded food composition databases that are highly representative of the Canadian packaged food supply is a strength of this study. Frequently updated, branded food composition data is needed to accurately monitor reformulation and food prices in the food supply. Generic food composition data, such as the Canadian Nutrient File which was last updated in 2015^(43)^, does not include food prices and averages nutrition information among similar foods, masking reformulation efforts by manufacturers at the product level. Our results are strengthened by the methods used to isolate the impact of reformulation on product prices. Matching products across years by product code (UPC or retailer-specific ID), retailer and container size controlled for price changes due to differences in retailer pricing (e.g. premium *v*. discount retailers, retailer type or retailer size^(44)^) and unit price differences driven by bulk buying, in which larger container sizes have lower unit prices^(45)^, or ‘shrinkflation’, when manufacturers decrease the container size but the unit price increases^(46,47)^. The impact of inflation on food prices was also removed as inflation was expected to impact reformulation groups equally, thereby cancelling out when comparing price changes across groups.

There are also limitations that should be considered. Results were presented by major food category and reformulation group; however, smaller sample sizes at the food category level and low frequencies of reformulation did not enable model fitting for all groups. The sample size was limited to products that had matching product codes (UPC or retailer-specific ID) in FLIP2017 and FLIP2020. If a product code was not available during sampling or changed between 2017 and 2020, the product would not have been included. Additionally, because of matching products by retailer, only products from the three retailers collected in FLIP2017 were included. Repeated collections of FLIP with the expanded number of retailers sampled in FLIP2020 would increase the sample size and enable more complete analysis at the major food category level and detailed analysis at the minor food category level.

Retailer location is also an important consideration. FLIP2017 and FLIP2020 were comparable as they were both collected using data from retailers in Toronto, Canada. However, results may differ if price data were collected from other locations across Canada, as food prices and changes in food prices over time vary by region^(48,49)^, due to regional variation in retailer pricing, such as the use of zone pricing^(50)^.

Notably, it was beyond the scope of this study to consider products that were introduced or removed from the food supply between 2017 and 2020. For example, rather than reformulating a current product, a manufacturer may have released a healthier version (e.g. a new lower sodium product line). Product turnover in the Canadian food supply and how it impacts the availability and price of healthier options is an area for further research. Additionally, these results only reflect price changes in the food supply between 2017 and 2020 and may differ across other time periods due to major global events (e.g. the COVID-19 pandemic, war in Ukraine, recent inflationary trends).

## Conclusions

Our findings show that reformulation was infrequent and bidirectional in the Canadian food supply between 2017 and 2020. This suggests that the voluntary policies during this time were not providing a strong incentive for manufacturers to reduce levels of nutrients of concern in their products. Stronger policy interventions are needed to make meaningful changes in the food supply. Overall, and in most food categories, there was no consistent association between reformulation and price changes, providing no evidence that reformulation impacts food prices. This suggests that food policies or regulations that incentivise reformulation may be implemented without resulting in increased prices for healthier foods.

## Supporting information

Ziraldo et al. supplementary material Ziraldo et al. supplementary material

## Data Availability

Data described in the manuscript and analytic code will be made available upon request, pending application and approval.

## Supplementary material

For supplementary material accompanying this paper visit https://doi.org/10.1017/S136898002400226X
