# Supplementary material for: Investigating reformulation in the Canadian food supply between 2017 and 2020 and its impact on food prices
Source: Public Health Nutr. 2024 Nov 22;27(1):e257. doi: 10.1017/S136898002400226X (PMC11705022; doi:10.1017/S136898002400226X)
Supplement: Ziraldo et al. supplementary material [file S136898002400226Xsup001.docx]

**Supplementary Table 1.** Descriptive statistics of food price change by retailer and by TRA food category in FLIP2017 and FLIP2020 matched products, n=5715 matched products.^†^

| **TRA Food Category** | **Year** | **All Retailers** | | |  | **Retailer A** | | |  | **Retailer B** | | |  | **Retailer C** | | |
| --- | --- | --- | --- | --- | --- | --- | --- | --- | --- | --- | --- | --- | --- | --- | --- | --- |
|  |  | **n** | **Mean (S.D.)** | **p** |  | **n** | **Mean (S.D.)** | **p** |  | **n** | **Mean (S.D.)** | **p** |  | **n** | **Mean (S.D.)** | **p** |
| A. Bakery | 2017 | 725 | 1.66 (1.10) | ** |  | 456 | 1.87 (1.17) | ns |  | 153 | 1.26 (0.99) | ** |  | 116 | 1.39 (0.67) | ns |
|  | 2020 | 725 | 1.68 (1.12) |  |  | 456 | 1.88 (1.19) |  |  | 153 | 1.29 (1.05) |  |  | 116 | 1.41 (0.66) |  |
| B. Beverages | 2017 | 291 | 1.74 (3.60) | *** |  | 169 | 2.43 (4.31) | *** |  | 90 | 1.01 (2.16) | ns |  | 32 | 0.13 (0.12) | ns |
|  | 2020 | 291 | 1.61 (3.35) |  |  | 169 | 2.21 (4.00) |  |  | 90 | 1.00 (2.12) |  |  | 32 | 0.13 (0.14) |  |
| C. Cereals and other grains | 2017 | 474 | 0.98 (0.66) | ns |  | 325 | 1.08 (0.67) | ns |  | 101 | 0.82 (0.67) | *** |  | 48 | 0.66 (0.38) | ** |
|  | 2020 | 474 | 0.98 (0.68) |  |  | 325 | 1.07 (0.69) |  |  | 101 | 0.86 (0.68) |  |  | 48 | 0.68 (0.41) |  |
| D. Dairy products and substitutes | 2017 | 563 | 1.75 (1.50) | * |  | 355 | 1.72 (1.52) | ns |  | 129 | 1.83 (1.69) | *** |  | 79 | 1.77 (0.99) | *** |
|  | 2020 | 563 | 1.75 (1.54) |  |  | 355 | 1.69 (1.56) |  |  | 129 | 1.87 (1.72) |  |  | 79 | 1.86 (1.04) |  |
| E. Desserts | 2017 | 274 | 1.11 (1.40) | ns |  | 157 | 1.43 (1.75) | ** |  | 99 | 0.65 (0.39) | *** |  | 18 | 0.81 (0.37) | ns |
|  | 2020 | 274 | 1.12 (1.35) |  |  | 157 | 1.39 (1.69) |  |  | 99 | 0.73 (0.44) |  |  | 18 | 0.85 (0.40) |  |
| F. Dessert toppings and fillings | 2017 | 36 | 1.19 (1.56) | ns |  | 28 | 1.27 (1.75) | ns |  | 4 | 1.09 (0.84) | ns |  | 4 | 0.78 (0.04) | ns |
|  | 2020 | 36 | 1.18 (1.57) |  |  | 28 | 1.25 (1.76) |  |  | 4 | 1.07 (0.85) |  |  | 4 | 0.78 (0.04) |  |
| G. Eggs and substitutes | 2017 | 23 | 0.72 (0.16) | * |  | 13 | 0.69 (0.15) | ** |  | 4 | 0.81 (0.08) | ns |  | 6 | 0.73 (0.22) | ns |
|  | 2020 | 23 | 0.76 (0.15) |  |  | 13 | 0.76 (0.14) |  |  | 4 | 0.80 (0.08) |  |  | 6 | 0.76 (0.23) |  |
| H. Fats and oils | 2017 | 316 | 1.14 (1.04) | ns |  | 164 | 1.33 (1.28) | * |  | 78 | 1.04 (0.65) | * |  | 74 | 0.81 (0.59) | ns |
|  | 2020 | 316 | 1.12 (0.99) |  |  | 164 | 1.28 (1.20) |  |  | 78 | 1.06 (0.66) |  |  | 74 | 0.82 (0.65) |  |
| I. Marine and fresh water animals | 2017 | 136 | 2.41 (1.40) | *** |  | 74 | 2.43 (1.46) | *** |  | 31 | 2.34 (1.44) | ** |  | 31 | 2.44 (1.24) | ** |
|  | 2020 | 136 | 2.61 (1.49) |  |  | 74 | 2.62 (1.51) |  |  | 31 | 2.56 (1.69) |  |  | 31 | 2.62 (1.24) |  |
| J. Fruit and fruit juices | 2017 | 361 | 0.68 (0.95) | ** |  | 233 | 0.64 (0.79) | ns |  | 64 | 0.98 (1.60) | * |  | 64 | 0.51 (0.41) | ** |
|  | 2020 | 361 | 0.70 (0.97) |  |  | 233 | 0.66 (0.82) |  |  | 64 | 1.00 (1.61) |  |  | 64 | 0.52 (0.41) |  |
| K. Legumes | 2017 | 94 | 0.40 (0.38) | *** |  | 44 | 0.49 (0.52) | ns |  | 28 | 0.34 (0.17) | *** |  | 22 | 0.31 (0.16) | *** |
|  | 2020 | 94 | 0.43 (0.44) |  |  | 44 | 0.50 (0.61) |  |  | 28 | 0.39 (0.21) |  |  | 22 | 0.36 (0.13) |  |
| L. Meat and substitutes | 2017 | 193 | 2.17 (1.57) | *** |  | 122 | 2.23 (1.78) | *** |  | 50 | 2.40 (1.18) | *** |  | 21 | 1.31 (0.48) | ns |
|  | 2020 | 193 | 2.31 (1.55) |  |  | 122 | 2.36 (1.74) |  |  | 50 | 2.63 (1.17) |  |  | 21 | 1.30 (0.48) |  |
| M. Miscellaneous | 2017 | 217 | 3.18 (3.10) | * |  | 152 | 3.53 (3.37) | *** |  | 53 | 2.41 (2.16) | ** |  | 12 | 2.10 (2.29) | ns |
|  | 2020 | 217 | 3.14 (3.17) |  |  | 152 | 3.38 (3.38) |  |  | 53 | 2.66 (2.58) |  |  | 12 | 2.12 (2.34) |  |
| N. Combination dishes | 2017 | 307 | 1.16 (0.61) | ns |  | 205 | 1.18 (0.57) | ** |  | 69 | 1.10 (0.74) | *** |  | 33 | 1.14 (0.53) | ** |
|  | 2020 | 307 | 1.15 (0.63) |  |  | 205 | 1.13 (0.57) |  |  | 69 | 1.21 (0.80) |  |  | 33 | 1.18 (0.54) |  |
| O. Nuts and seeds | 2017 | 110 | 2.86 (2.12) | ns |  | 61 | 2.91 (2.07) | * |  | 29 | 2.73 (2.07) | ns |  | 20 | 2.89 (2.45) | ns |
|  | 2020 | 110 | 2.75 (2.14) |  |  | 61 | 2.70 (2.11) |  |  | 29 | 2.76 (2.05) |  |  | 20 | 2.90 (2.47) |  |
| P. Potatoes, sweet potatoes and yams | 2017 | 43 | 0.58 (0.43) | ns |  | 30 | 0.65 (0.46) | ns |  | 10 | 0.44 (0.32) | ns |  | 3 | 0.28 (0.00) | ns |
|  | 2020 | 43 | 0.58 (0.44) |  |  | 30 | 0.64 (0.47) |  |  | 10 | 0.47 (0.41) |  |  | 3 | 0.33 (0.00) |  |
| Q. Salads | 2017 | 33 | 1.93 (0.81) | *** |  | 19 | 2.25 (0.78) | ** |  | 14 | 1.50 (0.64) | * |  | N/A | N/A | ns |
|  | 2020 | 33 | 2.06 (0.83) |  |  | 19 | 2.40 (0.81) |  |  | 14 | 1.59 (0.60) |  |  | N/A | N/A |  |
| R. Sauces, dips, gravies and condiments | 2017 | 416 | 1.79 (2.18) | ns |  | 275 | 2.20 (2.53) | ** |  | 77 | 1.26 (0.96) | ** |  | 64 | 0.70 (0.41) | ns |
|  | 2020 | 416 | 1.75 (2.08) |  |  | 275 | 2.11 (2.41) |  |  | 77 | 1.30 (0.96) |  |  | 64 | 0.71 (0.39) |  |
| S. Snacks | 2017 | 186 | 1.80 (1.29) | *** |  | 93 | 2.01 (1.43) | ** |  | 57 | 1.87 (1.26) | *** |  | 36 | 1.19 (0.53) | ** |
|  | 2020 | 186 | 1.89 (1.37) |  |  | 93 | 2.09 (1.58) |  |  | 57 | 1.97 (1.27) |  |  | 36 | 1.22 (0.51) |  |
| T. Soups | 2017 | 289 | 1.43 (2.49) | * |  | 223 | 1.56 (2.79) | ns |  | 31 | 1.18 (0.83) | ** |  | 35 | 0.76 (0.63) | ns |
|  | 2020 | 289 | 1.45 (2.60) |  |  | 223 | 1.59 (2.92) |  |  | 31 | 1.24 (0.83) |  |  | 35 | 0.79 (0.62) |  |
| U. Sugars and sweets | 2017 | 274 | 1.95 (1.24) | * |  | 183 | 2.16 (1.29) | ** |  | 59 | 1.68 (0.94) | ns |  | 32 | 1.27 (1.14) | ns |
|  | 2020 | 274 | 1.90 (1.16) |  |  | 183 | 2.08 (1.22) |  |  | 59 | 1.70 (0.93) |  |  | 32 | 1.22 (0.85) |  |
| V. Vegetables | 2017 | 354 | 0.79 (1.39) | * |  | 187 | 0.97 (1.71) | * |  | 94 | 0.68 (1.11) | *** |  | 73 | 0.47 (0.30) | *** |
|  | 2020 | 354 | 0.80 (1.48) |  |  | 187 | 0.96 (1.86) |  |  | 94 | 0.73 (1.11) |  |  | 73 | 0.50 (0.31) |  |

Abbreviations: FLIP, Food Label Information Program; Mean (S.D.), mean and standard deviation; n, sample size; ns, not significant; p, p-value; TRA, Table of Reference Amounts; UPC, universal product code.

^†^Food products matched by same ID (UPC and retailer-specific product number), retailer, and container size. Price per 100 g (or mL). Significance levels: *** p<0.001, ** p<0.01, * p<0.05, calculated by Wilcoxon signed-rank test.

**Supplementary Table 2.** Relationship between food price and calories, carbohydrate, protein and fat reformulation in matched products by TRA food category.^†^

| **TRA Food Category** | **Reformulation group^‡^** | **Calories** | | | |  | **Carbohydrates** | | | |  | **Protein** | | | |  | **Fat** | | | |
| --- | --- | --- | --- | --- | --- | --- | --- | --- | --- | --- | --- | --- | --- | --- | --- | --- | --- | --- | --- | --- |
|  |  | **n** | **%** | **β** | **95% CI** |  | **n** | **%** | **β** | **95% CI** |  | **n** | **%** | **β** | **95% CI** |  | **n** | **%** | **β** | **95% CI** |
| A. Bakery | Large decrease | 4 | 0.8 | — | — |  | 3 | 0.6 | — | — |  | 14 | 2.7 | -4.60 | -19.57, 10.37 |  | 25 | 4.7 | 4.46 | -7.04, 15.95 |
|  | Medium decrease | 30 | 5.7 | 2.39 | -8.00, 12.78 |  | 27 | 5.1 | -5.34 | -16.43, 5.76 |  | 5 | 0.9 | 0.89 | -21.93, 23.72 |  | 23 | 4.4 | 8.06 | -3.80, 19.92 |
|  | Little change | 465 | 88.1 | ref | ref |  | 473 | 89.6 | ref | ref |  | 491 | 93.0 | ref | ref |  | 447 | 84.7 | ref | ref |
|  | Medium increase | 27 | 5.1 | -5.76 | -17.13, 5.60 |  | 22 | 4.2 | 13.05* | 1.20, 24.9 |  | 4 | 0.8 | — | — |  | 14 | 2.7 | 10.63 | -5.70, 26.96 |
|  | Large increase | 2 | 0.4 | — | — |  | 3 | 0.6 | — | — |  | 14 | 2.7 | -1.37 | -17.53, 14.80 |  | 19 | 3.6 | 6.07 | -6.89, 19.04 |
| B. Beverages | Large decrease | 4 | 2.4 | — | — |  | 2 | 1.2 | — | — |  | 1 | 0.6 | — | — |  | 0 | 0.0 | — | — |
|  | Medium decrease | 2 | 1.2 | — | — |  | 6 | 3.6 | 13.85 | -29.34, 57.04 |  | 0 | 0.0 | — | — |  | 0 | 0.0 | — | — |
|  | Little change | 159 | 94.6 | ref | ref |  | 155 | 92.3 | ref | ref |  | 166 | 98.8 | ref | ref |  | 168 | 100.0 | ref | ref |
|  | Medium increase | 1 | 0.6 | — | — |  | 3 | 1.8 | — | — |  | 0 | 0.0 | — | — |  | 0 | 0.0 | — | — |
|  | Large increase | 2 | 1.2 | — | — |  | 2 | 1.2 | — | — |  | 1 | 0.6 | — | — |  | 0 | 0.0 | — | — |
| C. Cereals and other grains | Large decrease | 0 | 0.0 | — | — |  | 2 | 0.7 | — | — |  | 2 | 0.7 | — | — |  | 10 | 3.6 | 0.75 | -9.22, 12.03 |
|  | Medium decrease | 10 | 3.6 | -1.16 | -11.44, 10.47 |  | 2 | 0.7 | — | — |  | 0 | 0.0 | — | — |  | 1 | 0.4 | — | — |
|  | Little change | 257 | 92.8 | ref | ref |  | 268 | 96.8 | ref | ref |  | 255 | 92.1 | ref | ref |  | 242 | 87.4 | ref | ref |
|  | Medium increase | 9 | 3.2 | -3.85 | -15.29, 7.06 |  | 3 | 1.1 | — | — |  | 12 | 4.3 | -6.87 | -15.31, 4.89 |  | 1 | 0.4 | — | — |
|  | Large increase | 1 | 0.4 | — | — |  | 2 | 0.7 | — | — |  | 8 | 2.9 | 5.40 | -5.88, 17.81 |  | 23 | 8.3 | -3.29 | -9.96, 5.05 |
| D. Dairy products and substitutes | Large decrease | 3 | 0.7 | — | — |  | 18 | 4.3 | 0.57 | -10.99, 12.12 |  | 8 | 1.9 | -7.94 | -24.81, 8.92 |  | 7 | 1.7 | -1.01 | -19.04, 17.02 |
|  | Medium decrease | 13 | 3.1 | 3.59 | -9.75, 16.92 |  | 15 | 3.6 | 5.77 | -6.82, 18.37 |  | 6 | 1.4 | 0.58 | -18.84, 20.00 |  | 16 | 3.8 | 2.80 | -9.62, 15.21 |
|  | Little change | 383 | 91.0 | ref | ref |  | 367 | 87.2 | ref | ref |  | 401 | 95.2 | ref | ref |  | 374 | 88.8 | ref | ref |
|  | Medium increase | 18 | 4.3 | 7.53 | -3.87, 18.94 |  | 5 | 1.2 | 8.15 | -13.12, 29.41 |  | 3 | 0.7 | — | — |  | 11 | 2.6 | 15.01* | 0.48, 29.54 |
|  | Large increase | 4 | 1.0 | — | — |  | 16 | 3.8 | -1.46 | -13.56, 10.64 |  | 2 | 0.5 | — | — |  | 13 | 3.1 | -4.88 | -18.29, 8.52 |
| E. Desserts | Large decrease | 1 | 0.4 | — | — |  | 0 | 0.0 | — | — |  | 7 | 3.1 | -9.20 | -28.60, 10.56 |  | 3 | 1.3 | — | — |
|  | Medium decrease | 6 | 2.7 | 1.06 | -20.16, 22.01 |  | 9 | 4.0 | -0.77 | -18.20, 16.69 |  | 0 | 0.0 | — | — |  | 4 | 1.8 | — | — |
|  | Little change | 207 | 92.8 | ref | ref |  | 203 | 91.0 | ref | ref |  | 211 | 94.6 | ref | ref |  | 208 | 93.3 | ref | ref |
|  | Medium increase | 8 | 3.6 | -7.05 | -25.77, 11.42 |  | 7 | 3.1 | -3.47 | -23.39, 16.36 |  | 0 | 0.0 | — | — |  | 6 | 2.7 | -7.12 | -28.68, 13.77 |
|  | Large increase | 1 | 0.4 | — | — |  | 4 | 1.8 | — | — |  | 5 | 2.2 | 19.74 | -3.40, 43.08 |  | 2 | 0.9 | — | — |
| F. Dessert toppings and fillings | Large decrease | 0 | 0.0 | — | — |  | 0 | 0.0 | — | — |  | 1 | 3.7 | — | — |  | 2 | 7.4 | — | — |
|  | Medium decrease | 1 | 3.7 | — | — |  | 1 | 3.7 | — | — |  | 0 | 0.0 | — | — |  | 0 | 0.0 | — | — |
|  | Little change | 26 | 96.3 | ref | ref |  | 25 | 92.6 | ref | ref |  | 24 | 88.9 | ref | ref |  | 25 | 92.6 | ref | ref |
|  | Medium increase | 0 | 0.0 | — | — |  | 1 | 3.7 | — | — |  | 0 | 0.0 | — | — |  | 0 | 0.0 | — | — |
|  | Large increase | 0 | 0.0 | — | — |  | 0 | 0.0 | — | — |  | 2 | 7.4 | — | — |  | 0 | 0.0 | — | — |
| H. Fats and oils | Large decrease | 0 | 0.0 | — | — |  | 0 | 0.0 | — | — |  | 13 | 8.0 | 1.92 | -14.20, 22.67 |  | 0 | 0.0 | — | — |
|  | Medium decrease | 3 | 1.8 | — | — |  | 0 | 0.0 | — | — |  | 0 | 0.0 | — | — |  | 2 | 1.2 | — | — |
|  | Little change | 154 | 94.5 | ref | ref |  | 161 | 98.8 | ref | ref |  | 146 | 89.6 | ref | ref |  | 155 | 95.1 | ref | ref |
|  | Medium increase | 2 | 1.2 | — | — |  | 0 | 0.0 | — | — |  | 0 | 0.0 | — | — |  | 2 | 1.2 | — | — |
|  | Large increase | 4 | 2.5 | — | — |  | 2 | 1.2 | — | — |  | 4 | 2.5 | — | — |  | 4 | 2.5 | — | — |
| I. Marine and fresh water animals | Large decrease | 6 | 8.5 | 19.52 | -8.53, 49.64 |  | 1 | 1.4 | — | — |  | 2 | 2.8 | — | — |  | 5 | 7.0 | 19.17 | -11.31, 52.13 |
|  | Little decrease | 3 | 4.2 | — | — |  | 2 | 2.8 | — | — |  | 5 | 7.0 | -8.83 | -35.33, 22.00 |  | 1 | 1.4 | — | — |
|  | No change | 55 | 77.5 | ref | ref |  | 62 | 87.3 | ref | ref |  | 60 | 84.5 | ref | ref |  | 60 | 84.5 | ref | ref |
|  | Little increase | 4 | 5.6 | — | — |  | 0 | 0.0 | — | — |  | 1 | 1.4 | — | — |  | 0 | 0.0 | — | — |
|  | Large increase | 3 | 4.2 | — | — |  | 6 | 8.5 | -28.65 | -61.05, 4.96 |  | 3 | 4.2 | — | — |  | 5 | 7.0 | -28.39 | -63.29, 8.50 |
| J. Fruit and fruit juices | Large decrease | 9 | 3.8 | -0.50 | -6.63, 5.63 |  | 9 | 3.8 | 4.50 | -1.59, 10.58 |  | 12 | 5.1 | -3.68 | -9.04, 1.67 |  | 3 | 1.3 | — | — |
|  | Medium decrease | 13 | 5.5 | 5.07 | -0.17, 10.31 |  | 15 | 6.3 | -2.57 | -7.42, 2.28 |  | 0 | 0.0 | — | — |  | 1 | 0.4 | — | — |
|  | Little change | 209 | 88.2 | ref | ref |  | 199 | 84.0 | ref | ref |  | 213 | 89.9 | ref | ref |  | 227 | 95.8 | ref | ref |
|  | Medium increase | 2 | 0.8 | — | — |  | 8 | 3.4 | -3.51 | -9.99, 2.98 |  | 0 | 0.0 | — | — |  | 0 | 0.0 | — | — |
|  | Large increase | 4 | 1.7 | — | — |  | 6 | 2.5 | -4.03 | -11.51, 3.44 |  | 12 | 5.1 | -0.20 | -5.59, 5.20 |  | 6 | 2.5 | 10.75** | 3.36, 18.15 |
| K. Legumes | Large decrease | 0 | 0.0 | — | — |  | 0 | 0.0 | — | — |  | 0 | 0.0 | — | — |  | 2 | 8.0 | — | — |
|  | Medium decrease | 1 | 4.0 | — | — |  | 0 | 0.0 | — | — |  | 0 | 0.0 | — | — |  | 0 | 0.0 | — | — |
|  | Little change | 23 | 92.0 | ref | ref |  | 25 | 100.0 | ref | ref |  | 22 | 88.0 | ref | ref |  | 23 | 92.0 | ref | ref |
|  | Medium increase | 1 | 4.0 | — | — |  | 0 | 0.0 | — | — |  | 1 | 4.0 | — | — |  | 0 | 0.0 | — | — |
|  | Large increase | 0 | 0.0 | — | — |  | 0 | 0.0 | — | — |  | 2 | 8.0 | — | — |  | 0 | 0.0 | — | — |
| L. Meat and substitutes | Large decrease | 0 | 0.0 | — | — |  | 5 | 4.5 | -6.78 | -32.72, 19.38 |  | 5 | 4.5 | 13.58 | -11.44, 38.59 |  | 4 | 3.6 | — | — |
|  | Medium decrease | 8 | 7.1 | 8.57 | -11.60, 30.17 |  | 0 | 0.0 | — | — |  | 7 | 6.3 | 9.27 | -10.92, 32.61 |  | 7 | 6.3 | 14.31 | -8.17, 37.68 |
|  | Little change | 90 | 80.4 | ref | ref |  | 99 | 88.4 | ref | ref |  | 94 | 83.9 | ref | ref |  | 89 | 79.5 | ref | ref |
|  | Medium increase | 11 | 9.8 | 9.67 | -7.78, 28.41 |  | 0 | 0.0 | — | — |  | 2 | 1.8 | — | — |  | 4 | 3.6 | — | — |
|  | Large increase | 3 | 2.7 | — | — |  | 8 | 7.1 | 10.66 | -10.61, 32.64 |  | 4 | 3.6 | — | — |  | 8 | 7.1 | -8.74 | -29.55, 15.44 |
| M. Miscellaneous | Large decrease | 3 | 1.9 | — | — |  | 2 | 1.2 | — | — |  | 2 | 1.2 | — | — |  | 7 | 4.3 | 4.56 | -58.11, 62.71 |
|  | Medium decrease | 3 | 1.9 | — | — |  | 0 | 0.0 | — | — |  | 0 | 0.0 | — | — |  | 1 | 0.6 | — | — |
|  | Little change | 151 | 93.2 | ref | ref |  | 158 | 97.5 | ref | ref |  | 152 | 93.8 | ref | ref |  | 151 | 93.2 | ref | ref |
|  | Medium increase | 3 | 1.9 | — | — |  | 0 | 0.0 | — | — |  | 0 | 0.0 | — | — |  | 1 | 0.6 | — | — |
|  | Large increase | 2 | 1.2 | — | — |  | 2 | 1.2 | — | — |  | 8 | 4.9 | 42.38 | -10.36, 101.11 |  | 2 | 1.2 | — | — |
| N. Combination dishes | Large decrease | 6 | 2.8 | 14.39 | -1.42, 29.84 |  | 4 | 1.9 | — | — |  | 5 | 2.3 | 17.55* | 0.46, 34.34 |  | 14 | 6.5 | 11.57* | 1.05, 21.89 |
|  | Medium decrease | 6 | 2.8 | 3.12 | -12.58, 18.51 |  | 5 | 2.3 | -1.10 | -18.31, 16.15 |  | 5 | 2.3 | -1.31 | -18.41, 15.61 |  | 14 | 6.5 | 5.15 | -5.23, 15.73 |
|  | Little change | 186 | 86.9 | ref | ref |  | 185 | 86.4 | ref | ref |  | 180 | 84.1 | ref | ref |  | 171 | 79.9 | ref | ref |
|  | Medium increase | 10 | 4.7 | -2.77 | -15.00, 9.67 |  | 16 | 7.5 | 9.16 | -0.78, 19.44 |  | 13 | 6.1 | 1.90 | -9.54, 13.58 |  | 5 | 2.3 | -11.88 | -28.96, 6.19 |
|  | Large increase | 6 | 2.8 | 5.57 | -10.57, 22.17 |  | 4 | 1.9 | — | — |  | 11 | 5.1 | -2.46 | -14.38, 9.55 |  | 10 | 4.7 | -0.42 | -12.48, 12.05 |
| O. Nuts and seeds | Large decrease | 0 | 0.0 | — | — |  | 2 | 5.4 | — | — |  | 2 | 5.4 | — | — |  | 0 | 0.0 | — | — |
|  | Medium decrease | 10 | 27.0 | -5.91 | -19.81, 11.71 |  | 1 | 2.7 | — | — |  | 0 | 0.0 | — | — |  | 7 | 18.9 | -19.90* | -34.59, -4.61 |
|  | Little change | 27 | 73.0 | ref | ref |  | 30 | 81.1 | ref | ref |  | 32 | 86.5 | ref | ref |  | 30 | 81.1 | ref | ref |
|  | Medium increase | 0 | 0.0 | — | — |  | 0 | 0.0 | — | — |  | 2 | 5.4 | — | — |  | 0 | 0.0 | — | — |
|  | Large increase | 0 | 0.0 | — | — |  | 4 | 10.8 | — | — |  | 1 | 2.7 | — | — |  | 0 | 0.0 | — | — |
| P. Potatoes, sweet potatoes and yams | Large decrease | 1 | 3.0 | — | — |  | 2 | 6.1 | — | — |  | 2 | 6.1 | — | — |  | 3 | 9.1 | — | — |
|  | Medium decrease | 4 | 12.1 | — | — |  | 4 | 12.1 | — | — |  | 0 | 0.0 | — | — |  | 1 | 3.0 | — | — |
|  | Little change | 27 | 81.8 | ref | ref |  | 25 | 75.8 | ref | ref |  | 30 | 90.9 | ref | ref |  | 23 | 69.7 | ref | ref |
|  | Medium increase | 0 | 0.0 | — | — |  | 1 | 3.0 | — | — |  | 0 | 0.0 | — | — |  | 2 | 6.1 | — | — |
|  | Large increase | 1 | 3.0 | — | — |  | 1 | 3.0 | — | — |  | 1 | 3.0 | — | — |  | 4 | 12.1 | — | — |
| Q. Salads | Large decrease | 0 | 0.0 | — | — |  | 0 | 0.0 | — | — |  | 0 | 0.0 | — | — |  | 0 | 0.0 | — | — |
|  | Medium decrease | 0 | 0.0 | — | — |  | 0 | 0.0 | — | — |  | 1 | 5.0 | — | — |  | 2 | 10.0 | — | — |
|  | Little change | 16 | 80.0 | ref | ref |  | 16 | 80.0 | ref | ref |  | 18 | 90.0 | ref | ref |  | 16 | 80.0 | ref | ref |
|  | Medium increase | 2 | 10.0 | — | — |  | 3 | 15.0 | — | — |  | 0 | 0.0 | — | — |  | 2 | 10.0 | — | — |
|  | Large increase | 2 | 10.0 | — | — |  | 1 | 5.0 | — | — |  | 1 | 5.0 | — | — |  | 0 | 0.0 | — | — |
| R. Sauces, dips, gravies and condiments | Large decrease | 13 | 4.5 | 4.15 | -16.18, 28.20 |  | 18 | 6.2 | 4.05 | -14.01, 24.29 |  | 15 | 5.1 | 1.05 | -19.16, 21.93 |  | 6 | 2.1 | 3.24 | -29.32, 34.93 |
|  | Medium decrease | 4 | 1.4 | — | — |  | 5 | 1.7 | -9.10 | -44.35, 26.67 |  | 0 | 0.0 | — | — |  | 1 | 0.3 | — | — |
|  | Little change | 246 | 84.2 | ref | ref |  | 256 | 87.7 | ref | ref |  | 259 | 88.7 | ref | ref |  | 268 | 91.8 | ref | ref |
|  | Medium increase | 2 | 0.7 | — | — |  | 3 | 1.0 | — | — |  | 0 | 0.0 | — | — |  | 3 | 1.0 | — | — |
|  | Large increase | 27 | 9.2 | 8.61 | -7.02, 24.84 |  | 10 | 3.4 | -3.53 | -27.34, 23.42 |  | 18 | 6.2 | -12.25 | -30.70, 7.15 |  | 14 | 4.8 | 10.46 | -10.44, 32.34 |
| S. Snacks | Large decrease | 3 | 2.6 | — | — |  | 2 | 1.7 | — | — |  | 7 | 6.1 | 0.55 | -18.76, 19.86 |  | 5 | 4.3 | 11.35 | -11.45, 34.15 |
|  | Medium decrease | 5 | 4.3 | 1.14 | -21.98, 24.27 |  | 5 | 4.3 | -3.03 | -25.87, 19.82 |  | 3 | 2.6 | — | — |  | 5 | 4.3 | -4.24 | -27.43, 18.94 |
|  | Little change | 104 | 90.4 | ref | ref |  | 99 | 86.1 | ref | ref |  | 100 | 87.0 | ref | ref |  | 92 | 80.0 | ref | ref |
|  | Medium increase | 3 | 2.6 | — | — |  | 8 | 7.0 | 0.84 | -17.5, 19.18 |  | 3 | 2.6 | — | — |  | 9 | 7.8 | -9.99 | -27.32, 7.34 |
|  | Large increase | 0 | 0.0 | — | — |  | 1 | 0.9 | — | — |  | 2 | 1.7 | — | — |  | 4 | 3.5 | — | — |
| T. Soups | Large decrease | 6 | 2.8 | -2.65 | -18.77, 13.44 |  | 5 | 2.4 | -11.11 | -30.03, 7.80 |  | 11 | 5.2 | -2.67 | -15.06, 9.68 |  | 6 | 2.8 | 7.72 | -9.71, 25.15 |
|  | Medium decrease | 9 | 4.3 | 3.42 | -10.86, 17.68 |  | 11 | 5.2 | 3.88 | -9.39, 17.16 |  | 1 | 0.5 | — | — |  | 5 | 2.4 | 6.00 | -13.21, 25.21 |
|  | Little change | 178 | 84.4 | ref | ref |  | 180 | 85.3 | ref | ref |  | 185 | 87.7 | ref | ref |  | 179 | 84.8 | ref | ref |
|  | Medium increase | 11 | 5.2 | -4.33 | -17.32, 8.69 |  | 9 | 4.3 | 0.29 | -13.99, 14.57 |  | 1 | 0.5 | — | — |  | 2 | 0.9 | — | — |
|  | Large increase | 7 | 3.3 | -7.64 | -24.19, 8.94 |  | 6 | 2.8 | 5.69 | -11.98, 23.35 |  | 13 | 6.2 | -11.24 | -23.12, 0.70 |  | 19 | 9.0 | -2.42 | -12.78, 7.94 |
| U. Sugars and sweets | Large decrease | 6 | 3.4 | 16.87 | -7.76, 40.07 |  | 5 | 2.8 | -0.19 | -25.66, 23.53 |  | 4 | 2.2 | — | — |  | 2 | 1.1 | — | — |
|  | Medium decrease | 4 | 2.2 | — | — |  | 5 | 2.8 | 15.10 | -9.00, 38.71 |  | 0 | 0.0 | — | — |  | 1 | 0.6 | — | — |
|  | Little change | 166 | 92.7 | ref | ref |  | 167 | 93.3 | ref | ref |  | 167 | z | ref | ref |  | 170 | 95.0 | ref | ref |
|  | Medium increase | 2 | 1.1 | — | — |  | 0 | 0.0 | — | — |  | 0 | 0.0 | — | — |  | 1 | 0.6 | — | — |
|  | Large increase | 1 | 0.6 | — | — |  | 2 | 1.1 | — | — |  | 8 | 4.5 | -4.32 | -23.01, 15.50 |  | 5 | 2.8 | 25.53* | 2.08, 48.76 |
| V. Vegetables | Large decrease | 30 | 13.1 | 1.74 | -6.09, 9.58 |  | 17 | 7.4 | -0.75 | -10.62, 9.12 |  | 20 | 8.7 | 5.21 | -3.87, 14.28 |  | 4 | 1.7 | — | — |
|  | Medium decrease | 5 | 2.2 | -0.33 | -17.77, 17.12 |  | 6 | 2.6 | 1.82 | -14.25, 17.9 |  | 0 | 0.0 | — | — |  | 0 | 0.0 | — | — |
|  | Little change | 168 | 73.4 | ref | ref |  | 179 | 78.2 | ref | ref |  | 183 | 79.9 | ref | ref |  | 202 | 88.2 | ref | ref |
|  | Medium increase | 6 | 2.6 | 3.10 | -12.75, 18.95 |  | 7 | 3.1 | 4.11 | -10.75, 18.97 |  | 0 | 0.0 | — | — |  | 1 | 0.4 | — | — |
|  | Large increase | 20 | 8.7 | 0.18 | -8.82, 9.18 |  | 20 | 8.7 | -1.12 | -10.16, 7.91 |  | 26 | 11.4 | -0.78 | -8.72, 7.15 |  | 22 | 9.6 | -2.20 | -10.73, 6.32 |

Abbreviations: %, proportion of matches by reformulation group; β, beta coefficient ($\times$10-2); CI, confidence interval; n, sample size; ref, reference group; TRA, Table of Reference Amounts.

^†^Beta values for the effect size and confidence intervals were obtained by fitting mixed-effects models for price change and nutrition reformulation category per 100 g (or mL), adjusted for retailer, brand type, and container size, reference group is the little change group. All p-values were adjusted for multiple comparisons using the Benjamini-Hochberg procedure. Significance levels: ** p<0.01, * p<0.05. —, mixed effects model not fit due to small sample size (n<5). TRA food category G. Eggs and substitutes not included due to low sample size (n=9).

^‡^Products were categorized into five reformulation groups based on the magnitude and direction of calorie or nutrient changes per 100 g (or mL) between 2017 and 2020 using Health Canada’s labelling thresholds of 15% of the Daily Value (a lot) and 5% (a little) as cutoffs ^(30)^. The five reformulation groups were: 1) Large decrease (≥-15%), 2) Medium decrease (-5% to -14.9%), 3) Little change (-4.9% to +4.9%), 4) Medium increase (+5% to +14.9%), 5) Large increase (≥+15%).

**Supplementary Table 2 (continued).** Relationship between food price and saturated fat, sodium and sugar reformulation in matched products by TRA food category. ^†^

| **TRA Food Category** | **Reformulation group^‡^** | **Saturated Fat** | | | |  | **Sodium** | | | |  | **Sugar** | | | |
| --- | --- | --- | --- | --- | --- | --- | --- | --- | --- | --- | --- | --- | --- | --- | --- |
|  |  | **n** | **%** | **β** | **95% CI** |  | **n** | **%** | **β** | **95% CI** |  | **n** | **%** | **β** | **95% CI** |
| A. Bakery | Large decrease | 42 | 8.0 | -2.82 | -11.84, 6.20 |  | 36 | 6.8 | -6.45 | -16.21, 3.32 |  | 19 | 3.6 | 2.50 | -10.94, 15.93 |
|  | Medium decrease | 2 | 0.4 | — | — |  | 29 | 5.5 | -2.08 | -12.69, 8.54 |  | 11 | 2.1 | -7.97 | -24.74, 8.80 |
|  | Little change | 457 | 86.6 | ref | ref |  | 419 | 79.4 | ref | ref |  | 455 | 86.2 | ref | ref |
|  | Medium increase | 4 | 0.8 | — | — |  | 24 | 4.5 | -6.27 | -17.79, 5.26 |  | 25 | 4.7 | -8.02 | -19.18, 3.13 |
|  | Large increase | 23 | 4.4 | 6.82 | -5.26, 18.89 |  | 20 | 3.8 | 11.84 | -0.54, 24.21 |  | 18 | 3.4 | -1.43 | -14.78, 11.91 |
| B. Beverages | Large decrease | 0 | 0.0 | — | — |  | 7 | 4.2 | 7.30 | -33.23, 47.84 |  | 4 | 2.4 | — | — |
|  | Medium decrease | 0 | 0.0 | — | — |  | 4 | 2.4 | — | — |  | 4 | 2.4 | — | — |
|  | Little change | 167 | 99.4 | ref | ref |  | 146 | 86.9 | ref | ref |  | 155 | 92.3 | ref | ref |
|  | Medium increase | 1 | 0.6 | — | — |  | 1 | 0.6 | — | — |  | 3 | 1.8 | — | — |
|  | Large increase | 0 | 0.0 | — | — |  | 10 | 6.0 | 15.92 | -16.97, 48.81 |  | 2 | 1.2 | — | — |
| C. Cereals and other grains | Large decrease | 25 | 9.0 | 1.77 | -5.37, 9.11 |  | 12 | 4.3 | 13.57** | 4.15, 22.99 |  | 3 | 1.1 | — | — |
|  | Medium decrease | 0 | 0.0 | — | — |  | 3 | 1.1 | — | — |  | 2 | 0.7 | — | — |
|  | Little change | 226 | 81.6 | ref | ref |  | 246 | 88.8 | ref | ref |  | 247 | 89.2 | ref | ref |
|  | Medium increase | 0 | 0.0 | — | — |  | 4 | 1.4 | — | — |  | 2 | 0.7 | — | — |
|  | Large increase | 26 | 9.4 | -4.34 | -10.63, 3.58 |  | 12 | 4.3 | -12.89** | -22.32, -3.45 |  | 23 | 8.3 | -0.24 | -7.53, 6.87 |
| D. Dairy products and substitutes | Large decrease | 7 | 1.7 | -0.18 | -18.06, 17.69 |  | 16 | 3.8 | 3.54 | -8.61, 15.70 |  | 15 | 3.6 | 7.48 | -5.19, 20.16 |
|  | Medium decrease | 3 | 0.7 | — | — |  | 32 | 7.6 | -3.45 | -12.43, 5.54 |  | 3 | 0.7 | — | — |
|  | Little change | 372 | 88.4 | ref | ref |  | 329 | 78.1 | ref | ref |  | 392 | 93.1 | ref | ref |
|  | Medium increase | 6 | 1.4 | 21.12* | 1.77, 40.47 |  | 22 | 5.2 | -2.27 | -12.76, 8.22 |  | 4 | 1.0 | — | — |
|  | Large increase | 33 | 7.8 | -7.80 | -16.47, 0.87 |  | 22 | 5.2 | 8.41 | -2.03, 18.85 |  | 7 | 1.7 | 5.99 | -11.99, 23.97 |
| E. Desserts | Large decrease | 4 | 1.8 | — | — |  | 8 | 3.6 | -6.89 | -25.29, 11.47 |  | 1 | 0.4 | — | — |
|  | Medium decrease | 5 | 2.2 | -9.72 | -19.57, 27.26 |  | 8 | 3.6 | 2.24 | -15.94, 20.96 |  | 8 | 3.6 | 0.21 | -18.09, 18.22 |
|  | Little change | 201 | 90.1 | ref | ref |  | 189 | 84.8 | ref | ref |  | 207 | 92.8 | ref | ref |
|  | Medium increase | 5 | 2.2 | 2.84 | -20.57, 25.73 |  | 8 | 3.6 | -14.69 | -32.90, 3.81 |  | 4 | 1.8 | — | — |
|  | Large increase | 8 | 3.6 | 13.87 | -4.75, 32.05 |  | 10 | 4.5 | -2.39 | -18.67, 14.29 |  | 3 | 1.3 | — | — |
| F. Dessert toppings and fillings | Large decrease | 2 | 7.4 | — | — |  | 0 | 0.0 | — | — |  | 0 | 0.0 | — | — |
|  | Medium decrease | 0 | 0.0 | — | — |  | 0 | 0.0 | — | — |  | 2 | 7.4 | — | — |
|  | Little change | 25 | 92.6 | ref | ref |  | 26 | 96.3 | ref | ref |  | 25 | 92.6 | ref | ref |
|  | Medium increase | 0 | 0.0 | — | — |  | 1 | 3.7 | — | — |  | 0 | 0.0 | — | — |
|  | Large increase | 0 | 0.0 | — | — |  | 0 | 0.0 | — | — |  | 0 | 0.0 | — | — |
| H. Fats and oils | Large decrease | 5 | 3.1 | -11.44 | -39.28, 19.48 |  | 6 | 3.7 | -8.39 | -31.94, 16.98 |  | 0 | 0.0 | — | — |
|  | Medium decrease | 3 | 1.8 | — | — |  | 1 | 0.6 | — | — |  | 0 | 0.0 | — | — |
|  | Little change | 144 | 88.3 | ref | ref |  | 147 | 90.2 | ref | ref |  | 158 | 96.9 | ref | ref |
|  | Medium increase | 0 | 0.0 | — | — |  | 3 | 1.8 | — | — |  | 0 | 0.0 | — | — |
|  | Large increase | 11 | 6.7 | 1.64 | -16.36, 23.60 |  | 6 | 3.7 | -53.13*** | -77.34, -29.02 |  | 5 | 3.1 | -4.69 | -31.68, 25.71 |
| I. Marine and fresh water animals | Large decrease | 3 | 4.2 | — | — |  | 1 | 1.4 | — | — |  | 4 | 5.6 | — | — |
|  | Medium decrease | 0 | 0.0 | — | — |  | 3 | 4.2 | — | — |  | 0 | 0.0 | — | — |
|  | Little change | 66 | 93.0 | ref | ref |  | 60 | 84.5 | ref | ref |  | 63 | 88.7 | ref | ref |
|  | Medium increase | 0 | 0.0 | — | — |  | 2 | 2.8 | — | — |  | 0 | 0.0 | — | — |
|  | Large increase | 2 | 2.8 | — | — |  | 5 | 7.0 | -21.94 | -51.08, 8.98 |  | 4 | 5.6 | — | — |
| J. Fruit and fruit juices | Large decrease | 5 | 2.1 | -6.79 | -14.91, 1.32 |  | 14 | 5.9 | 2.33 | -2.69, 7.34 |  | 16 | 6.8 | 0.39 | -4.49, 5.26 |
|  | Medium decrease | 0 | 0.0 | — | — |  | 6 | 2.5 | 0.16 | -7.70, 8.01 |  | 7 | 3.0 | 0.27 | -6.66, 7.20 |
|  | Little change | 230 | 97.0 | ref | ref |  | 200 | 84.4 | ref | ref |  | 195 | 82.3 | ref | ref |
|  | Medium increase | 0 | 0.0 | — | — |  | 1 | 0.4 | — | — |  | 8 | 3.4 | 7.53* | 0.95, 14.12 |
|  | Large increase | 2 | 0.8 | — | — |  | 16 | 6.8 | -0.52 | -5.25, 4.20 |  | 11 | 4.6 | 0.51 | -5.04, 6.06 |
| K. Legumes | Large decrease | 1 | 4.0 | — | — |  | 0 | 0.0 | — | — |  | 0 | 0.0 | — | — |
|  | Medium decrease | 0 | 0.0 | — | — |  | 0 | 0.0 | — | — |  | 0 | 0.0 | — | — |
|  | Little change | 23 | 92.0 | ref | ref |  | 22 | 88.0 | ref | ref |  | 23 | 92.0 | ref | ref |
|  | Medium increase | 0 | 0.0 | — | — |  | 0 | 0.0 | — | — |  | 0 | 0.0 | — | — |
|  | Large increase | 1 | 4.0 | — | — |  | 3 | 12.0 | — | — |  | 2 | 8.0 | — | — |
| L. Meat and substitutes | Large decrease | 9 | 8.0 | -27.01** | -45.99, -5.60 |  | 6 | 5.4 | 9.40 | -12.81, 31.61 |  | 7 | 6.3 | -18.27 | -39.79, 3.42 |
|  | Medium decrease | 0 | 0.0 | — | — |  | 5 | 4.5 | 35.57** | 11.57, 59.58 |  | 0 | 0.0 | — | — |
|  | Little change | 92 | 82.1 | ref | ref |  | 87 | 77.7 | ref | ref |  | 102 | 91.1 | ref | ref |
|  | Medium increase | 3 | 2.7 | — | — |  | 7 | 6.3 | -33.15** | -53.80, -12.50 |  | 1 | 0.9 | — | — |
|  | Large increase | 8 | 7.1 | 1.05 | -20.02, 30.70 |  | 7 | 6.3 | -24.90* | -45.58, -4.23 |  | 2 | 1.8 | — | — |
| M. Miscellaneous | Large decrease | 8 | 4.9 | 44.03 | -14.07, 98.68 |  | 3 | 1.9 | — | — |  | 1 | 0.6 | — | — |
|  | Medium decrease | 1 | 0.6 | — | — |  | 5 | 3.1 | 27.20 | -40.44, 100.44 |  | 1 | 0.6 | — | — |
|  | Little change | 150 | 92.6 | ref | ref |  | 146 | 90.1 | ref | ref |  | 156 | 96.3 | ref | ref |
|  | Medium increase | 0 | 0.0 | — | — |  | 2 | 1.2 | — | — |  | 1 | 0.6 | — | — |
|  | Large increase | 3 | 1.9 | — | — |  | 6 | 3.7 | 25.72 | -40.74, 88.11 |  | 3 | 1.9 | — | — |
| N. Combination dishes | Large decrease | 22 | 10.3 | 11.02* | 2.57, 19.44 |  | 6 | 2.8 | 1.49 | -14.21, 17.47 |  | 20 | 9.3 | 6.85 | -2.34, 16.07 |
|  | Medium decrease | 4 | 1.9 | — | — |  | 17 | 7.9 | 4.13 | -5.33, 13.63 |  | 1 | 0.5 | — | — |
|  | Little change | 164 | 76.6 | ref | ref |  | 170 | 79.4 | ref | ref |  | 162 | 75.7 | ref | ref |
|  | Medium increase | 4 | 1.9 | — | — |  | 12 | 5.6 | 12.83* | 1.69, 24.21 |  | 2 | 0.9 | — | — |
|  | Large increase | 20 | 9.3 | 0.70 | -8.13, 9.88 |  | 9 | 4.2 | 3.83 | -9.47, 17.15 |  | 29 | 13.6 | 1.07 | -6.83, 9.35 |
| O. Nuts and seeds | Large decrease | 7 | 18.9 | -0.83 | -13.30, 19.06 |  | 3 | 8.1 | — | — |  | 2 | 5.4 | — | — |
|  | Medium decrease | 1 | 2.7 | — | — |  | 4 | 10.8 | — | — |  | 0 | 0.0 | — | — |
|  | Little change | 26 | 70.3 | ref | ref |  | 27 | 73.0 | ref | ref |  | 34 | 91.9 | ref | ref |
|  | Medium increase | 0 | 0.0 | — | — |  | 0 | 0.0 | — | — |  | 0 | 0.0 | — | — |
|  | Large increase | 3 | 8.1 | — | — |  | 3 | 8.1 | — | — |  | 1 | 2.7 | — | — |
| P. Potatoes, sweet potatoes and yams | Large decrease | 3 | 9.1 | — | — |  | 7 | 21.2 | -0.60 | -4.56, 3.35 |  | 0 | 0.0 | — | — |
|  | Medium decrease | 0 | 0.0 | — | — |  | 1 | 3.0 | — | — |  | 0 | 0.0 | — | — |
|  | Little change | 27 | 81.8 | ref | ref |  | 22 | 66.7 | ref | ref |  | 32 | 97.0 | ref | ref |
|  | Medium increase | 0 | 0.0 | — | — |  | 2 | 6.1 | — | — |  | 0 | 0.0 | — | — |
|  | Large increase | 3 | 9.1 | — | — |  | 1 | 3.0 | — | — |  | 1 | 3.0 | — | — |
| Q. Salads | Large decrease | 0 | 0.0 | — | — |  | 0 | 0.0 | — | — |  | 0 | 0.0 | — | — |
|  | Medium decrease | 0 | 0.0 | — | — |  | 0 | 0.0 | — | — |  | 0 | 0.0 | — | — |
|  | Little change | 18 | 90.0 | ref | ref |  | 15 | 75.0 | ref | ref |  | 16 | 80.0 | ref | ref |
|  | Medium increase | 0 | 0.0 | — | — |  | 3 | 15.0 | — | — |  | 1 | 5.0 | — | — |
|  | Large increase | 2 | 10.0 | — | — |  | 2 | 10.0 | — | — |  | 3 | 15.0 | — | — |
| R. Sauces, dips, gravies and condiments | Large decrease | 5 | 1.7 | 6.16 | -27.74, 42.24 |  | 9 | 3.1 | 4.30 | -21.80, 31.17 |  | 14 | 4.8 | 16.17 | -4.53, 38.07 |
|  | Medium decrease | 0 | 0.0 | — | — |  | 19 | 6.5 | 16.05 | -1.86, 35.25 |  | 1 | 0.3 | — | — |
|  | Little change | 270 | 92.5 | ref | ref |  | 243 | 83.2 | ref | ref |  | 259 | 88.7 | ref | ref |
|  | Medium increase | 0 | 0.0 | — | — |  | 8 | 2.7 | -34.93* | -62.25, -7.10 |  | 2 | 0.7 | — | — |
|  | Large increase | 17 | 5.8 | 4.31 | -14.11, 24.80 |  | 13 | 4.5 | -6.23 | -28.26, 15.85 |  | 16 | 5.5 | 0.96 | -18.35, 22.19 |
| S. Snacks | Large decrease | 10 | 8.7 | 4.99 | -11.34, 21.32 |  | 10 | 8.7 | -8.98 | -25.48, 7.52 |  | 8 | 7.0 | -0.52 | -18.96, 17.91 |
|  | Medium decrease | 1 | 0.9 | — | — |  | 2 | 1.7 | — | — |  | 0 | 0.0 | — | — |
|  | Little change | 99 | 86.1 | ref | ref |  | 93 | 80.9 | ref | ref |  | 105 | 91.3 | ref | ref |
|  | Medium increase | 0 | 0.0 | — | — |  | 5 | 4.3 | 4.09 | -19.07, 27.26 |  | 1 | 0.9 | — | — |
|  | Large increase | 5 | 4.3 | -0.65 | -23.25, 21.95 |  | 5 | 4.3 | 11.05 | -12.03, 34.13 |  | 1 | 0.9 | — | — |
| T. Soups | Large decrease | 5 | 2.4 | 0.11 | -18.81, 19.03 |  | 4 | 1.9 | — | — |  | 22 | 10.4 | -3.01 | -12.56, 6.55 |
|  | Medium decrease | 2 | 0.9 | — | — |  | 12 | 5.7 | 5.33 | -7.31, 17.97 |  | 2 | 0.9 | — | — |
|  | Little change | 188 | 89.1 | ref | ref |  | 177 | 83.9 | ref | ref |  | 173 | 82.0 | ref | ref |
|  | Medium increase | 0 | 0.0 | — | — |  | 11 | 5.2 | -3.45 | -16.43, 9.53 |  | 2 | 0.9 | — | — |
|  | Large increase | 16 | 7.6 | 4.05 | -7.01, 15.11 |  | 7 | 3.3 | -8.34 | -23.78, 7.11 |  | 12 | 5.7 | 6.13 | -6.37, 18.63 |
| U. Sugars and sweets | Large decrease | 1 | 0.6 | — | — |  | 7 | 3.9 | 14.16 | -5.93, 34.35 |  | 6 | 3.4 | 13.04 | -9.56, 34.43 |
|  | Medium decrease | 2 | 1.1 | — | — |  | 3 | 1.7 | — | — |  | 7 | 3.9 | -4.01 | -24.61, 16.09 |
|  | Little change | 170 | 95.0 | ref | ref |  | 153 | 85.5 | ref | ref |  | 158 | 88.3 | ref | ref |
|  | Medium increase | 1 | 0.6 | — | — |  | 1 | 0.6 | — | — |  | 6 | 3.4 | -11.00 | -32.66, 10.55 |
|  | Large increase | 5 | 2.8 | 17.73 | -5.17, 41.66 |  | 15 | 8.4 | 12.72 | -1.23, 26.78 |  | 2 | 1.1 | — | — |
| V. Vegetables | Large decrease | 4 | 1.7 | — | — |  | 33 | 14.4 | 7.23 | -0.27, 14.73 |  | 19 | 8.3 | -0.29 | -9.56, 8.98 |
|  | Medium decrease | 0 | 0.0 | — | — |  | 11 | 4.8 | -2.20 | -14.09, 9.69 |  | 1 | 0.4 | — | — |
|  | Little change | 218 | 95.2 | ref | ref |  | 160 | 69.9 | ref | ref |  | 189 | 82.5 | ref | ref |
|  | Medium increase | 0 | 0.0 | — | — |  | 5 | 2.2 | -2.85 | -19.97, 14.27 |  | 2 | 0.9 | — | — |
|  | Large increase | 7 | 3.1 | -0.28 | -14.84, 14.28 |  | 20 | 8.7 | -0.82 | -9.81, 8.17 |  | 18 | 7.9 | 3.60 | -5.85, 13.04 |

Abbreviations: %, proportion of matches by reformulation group; β, beta coefficient ($\times$10^-2^); CI, confidence interval; n, sample size; ref, reference group; TRA, Table of Reference Amounts.

^†^Beta values for the effect size and confidence intervals were obtained by fitting mixed-effects models for price change and nutrition reformulation category per 100 g (or mL), adjusted for retailer, brand type, and container size, reference group is the little change group. All p-values were adjusted for multiple comparisons using the Benjamini-Hochberg procedure. Significance levels: *** p<0.001, ** p<0.01, * p<0.05. —, mixed effects model not fit due to small sample size (n<5). TRA food category G. Eggs and substitutes not included due to low sample size (n=9).

^‡^Products were categorized into five reformulation groups based on the magnitude and direction of calorie or nutrient changes per 100 g (or mL) between 2017 and 2020 using Health Canada’s labelling thresholds of 15% of the Daily Value (a lot) and 5% (a little) as cutoffs ^(30)^. The five reformulation groups were: 1) Large decrease (≥-15%), 2) Medium decrease (-5% to -14.9%), 3) Little change (-4.9% to +4.9%), 4) Medium increase (+5% to +14.9%), 5) Large increase (≥+15%).
